# Microwave-Assisted, One-Pot Synthesis of Doxycycline under Heterogeneous Catalysis in Water

**DOI:** 10.3390/antibiotics10091084

**Published:** 2021-09-08

**Authors:** Fabio Bucciol, Elia Maffeis, Emanuela Calcio Gaudino, László Jicsinszky, Silvia Tagliapietra, Alessandro Barge, Cristina Prandi, Giancarlo Cravotto

**Affiliations:** 1 Department of Drug Science and Technology, University of Turin, Via P. Giuria 9, 10125 Turin, Italy; fabio.bucciol@unito.it (F.B.); emanuela.calcio@unito.it (E.C.G.); laszlo.jicsinszky@unito.it (L.J.); silvia.tagliapietra@unito.it (S.T.); alessandro.barge@unito.it (A.B.); 2 Huvepharma Italia Srl, Via Roberto Lepetit, 142, 12075 Garessio (CN), Italy; elia.maffeis@unito.it; 3 Department of Chemistry, University of Turin, Via P. Giuria 7, 10125 Turin, Italy; cristina.prandi@unito.it

**Keywords:** doxycycline, microwave-assisted reaction, cyclodextrins complexation, rhodium catalyst, stereoselective synthesis

## Abstract

The selective synthesis of active pharmaceutical molecules is a challenging issue, particularly when attempting to make the reactions even more sustainable. The present work focuses on the microwave-assisted hydrogenolysis of oxytetracycline to selectively produce α-doxycycline. Although the combination of microwave irradiation and a heterogeneous rhodium catalyst provided good conversions, the selective synthesis of active α-doxycycline was only achieved when an oxytetracycline-cyclodextrin complex was used as the starting material, giving the desired product at 34.0% yield in a one-step reaction under very mild conditions.

## 1. Introduction

Tetracyclines are one of the most widely used classes of antibiotics [1] for human and veterinary use [2]. First isolated from *Streptomyces aureofaciens* in the 1940s, they derive their name from tetracycline, which is the simplest of these molecules that still retain antibiotic functions, although it was not the first to be isolated [3]. First-generation tetracyclines, such as chlortetracycline and oxytetracycline, naturally occur in soil bacteria colonies [4,5,6]. Second-generation tetracyclines are synthetic or semi-synthetic drugs that are often produced via the modification of natural tetracyclines. They show improved activity, stability and reduce the insurgence of bacterial resistance [7,8,9]. In particular, doxycycline, 4-dimethylamino-1,4,4a,5,5a,6,11,12a-octahydro-3,5,10,12,12a-pentahydroxy-6-methyl-1,11-dioxo-2,naphthacencarboxamide, is one of the most commonly used semi-synthetic derivatives of tetracycline [10]. Doxycycline is an isomer of tetracycline that differs only in the position of one hydroxyl group. Doxycycline can be formally viewed as the result of the transfer of the C_6_ hydroxyl group of tetracycline to C_5_. It is bacteriostatic and acts by inhibiting bacterial protein synthesis via the disruption of transfer and messenger RNA at ribosomal sites [11]. Its synthesis was first reported by Pfizer [12], and it is nowadays industrially produced starting from oxytetracycline, having methacycline as an intermediate. Another early route for this synthesis was reported and patented by Jensen and McCormick in 1962 [13] and involved the hydrogenolysis of oxytetracycline over an Rh/C 5 wt% catalyst under H_2_ pressure. While this direct conversion over a heterogeneous catalyst was appealing, it made use of dimethylformamide (DMF) and water 1:1 mixture and involved 16 h of reaction at room temperature. Nevertheless, few improvements have been made to this process since then; this is mainly because of the high cost of rhodium and the low doxycycline yields, with 6-epi-doxycycline being a common secondary product [14]. Indeed, C6 hydrogenolysis involves a stereocenter and can result in two epimers: the α (common doxycycline, pharmaceutically active) and the β (6-epi-doxycycline) forms. β epimer formation may be favored when a heterogeneous catalyst is used because of the substrate’s skeletal configuration, which leads to it approaching the catalyst from the side of the methyl substituent [15]. More recent work involved the hydrogenation of methacycline over a Pd/C 0.4 wt% catalyst that was poisoned with a thiourea/quinoline mixture [16]. The mixture coordinates the Pd active sites, creating a steric hindrance that pushes the reaction towards the desired product. This approach led to 94.5% enantioselectivity towards α-doxycycline and conversion of 90.7%. The introduction of chirality in drugs during their synthesis is of crucial importance. Over the past few years, the new opportunities offered by homogeneous transition metal catalysis have been realized [17].

Many authors have opted for the use of homogeneous Rh(I) and Rh(III) catalysts instead, controlling the enantioselectivity of the reaction via the steric hindrance of the organic ligands [13,18]. The total synthesis of doxycycline, starting from simple building blocks, is also possible, but generally involves several steps that lower the yield. One example is the enantioselective synthesis reported by Charest et al., which indeed led to the α form of doxycycline, but in 18 steps with a yield of 8.3% [19].

The present work focuses on the hydrogenolysis of oxytetracycline via the screening of different supported Rh catalysts. The objective was not only the synthesis of α-doxycycline but also the improvement of the conditions found in the literature to create a greener protocol. For this reason, we also compared conventional and microwave (MW) heating. MW was used to reduce both the reaction time and the amount of catalyst needed, exploiting their fast and intense activation of metallic nanoparticles [20,21]. MW is known for its fast-heating rate and the creation of hotspots and inverse thermal gradients [22,23,24]. The use of a heterogeneous catalyst creates synergistic effects with MW and allows the solid to be easily recovered and reused [25]. Furthermore, once we established which heating technique works best, cyclodextrins (CDs) were used as complexing agents for the starting oxytetracycline to test whether it is possible to influence the enantioselectivity of the reaction. CDs are cyclic oligosaccharides that are made up of glucopyranose units connected by α-1,4 glycosidic bonds. They are non-toxic and widely used even in food applications [26]. CDs are the one of most used chiral selectors in capillary electrophoresis and their exclusive structures, involving a truncated cone shape cavity with a hydrophilic external surface and a hydrophobic internal surface, making them effective for separation purposes [27,28,29]. Indeed, CDs can form host–guest complexes with organic molecules. Host–guest complexes are molecular aggregates that are stabilized via non-covalent bonds (for example, van der Waals, hydrogen bonds, and hydrophobic interactions) that can be easily broken under mild conditions (using an organic solvent such as methanol or ethanol). Tetracycline supramolecular complexes with CDs have previously been investigated [30] for use in analytical and biological applications, but, to the best of our knowledge, no synthetic investigations on similar supramolecular complexes (involving tetracyclines and CDs) have been performed so far. In the present work, the inclusion process of oxytetracycline with CDs has been investigated, for the first time, for the selective MW-assisted synthesis of α-doxycycline under heterogeneous catalysis.

## 2. Materials and Methods

All organic reagents, catalysts, and solvents were purchased from Sigma Aldrich (Sigma Aldrich, Darmstadt, Germany) and used without further purification. The CDs were purchased from Wacker Chemie, Munich, Germany. MW-assisted reactions were carried out in a SynthWAVE reactor (MLS GmbH, Leutkirch im Allgäu, Germany; Milestone Srl, Bergamo, Italy), which housed a closed MW-cavity. Conventional reactions were performed in a 300 mL PolyBlock stainless steel reactor.

HPLC-DAD analysis was performed using an HPLC system (Waters Corp., Milford, MA, USA) coupled with a diode array detector (UV/DAD, Waters Corp., Milford, MA, USA) and an automatic sampler (Waters Corp., Milford, MA, USA). A Gemini NXC18 column (250 mm, 4.6 mm, 5 μm; Phenomenex, Torrance, CA, USA) was used with gradient elution and UV-DAD acquisitions at 270 and 360 nm.

A Fraction Link separation module, equipped with Waters Micromass ZQ Mass Spectrometer was used to identify the final product (see Supplementary Materials).

NMR spectra were recorded on a JEOL ECZR 600 spectrometer, operating at 14T, at 25 °C in an appropriate deuterated solvent; chemical shifts were referenced using the residual solvent proton or carbon resonances. Full assignment was obtained using COSY, ^1^H-^13^C HMQC, ^1^H-^13^C HMBC 2D-spectroscopies (see Supplementary Materials).

### 2.1. General Procedure for the Conventional and MW-Assisted Synthesis of Doxycycline

The 300 mL PolyBlock stainless steel reactor used for the conventional heating tests was equipped with temperature and pressure control and a PTFE stirrer. The reactor could operate up to 100 bar and 250 °C. A sampling valve on the bottom of the reactor was directly connected to the cooling chamber.

MW-assisted reactions were carried out in a SynthWAVE reactor, which is a multimode MW system, equipped with multiple gas inlets, able to operate up to 300 °C and 199 bars, both on small and large scales (from 2 to 600 mL). Integrated sensors continuously monitor the internal pressure and temperature allowing the software to adjust, in real-time, the applied MW power to follow a predefined temperature profile.

In a typical experiment both with MW and with conventional heating, oxytetracycline (0.5 mmol) was dissolved in 2 mL of solvent (DMF/H_2_O 1:1, MeOH, or H_2_O) in the presence of 50 mg of supported Rh catalysts (5 wt.%). The reactions were performed under magnetic stirring (400 rpm) at a fixed temperature and for a predefined time (see Table 1) under H_2_ pressure of 5 or 10 bar.

### 2.2. General Procedure for Oxytetracycline-CD Complexation and Further Hydrogenolysis Reaction

CDs were used as chiral selectors. Three different CDs were considered: βCD, randomly methylated βCD (RAMEB), and γCD. Water was used as a solvent to favor CD dissolution and avoid any possible competition in the complexation reaction. HCl 0.01 M was used to allow oxytetracycline dissolution.

**Figure 1:**
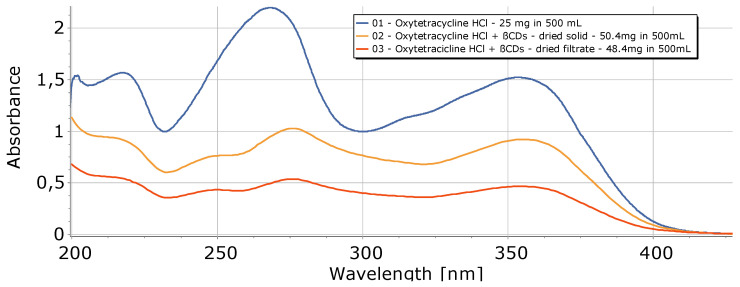
Oxytetracycline/β-CD complex UV-Vis analyses.

Complexation was performed with a 1:1 molar ratio of CDs (5.50 g) to oxytetracycline (1.92 g) in water. CDs were not dried before use and a 14 wt% (βCD) and 10 wt% (γCD) of water were considered. HCl 0.001 M was added to solubilize the cycline. The solution was then stirred for 12 h and filtered. Both RAMEB and γCD resulted in a homogeneous solution that was collected and freeze-dried to obtain a yellow crystalline solid. The βCD resulted in a precipitate that was collected after filtration and dried in a vacuum (3.10 g). The solution was freeze-dried, obtaining 4.32 g solid. UV-Vis analysis of the two solid fractions indicates a content of 30% w/w of oxytetracycline for the first solid and 25% w/w for the second one (Figure 1). The UV-Vis spectra were visualized via SpectraGryph 1.2 software.

The so-formed complexes (150 mg) were hydrogenated in water (2 mL) under 5 bar of H_2_ using an Rh/AC or Rh/Al_2_O_3_ 5 wt % commercial catalyst in a 1:1 weight ratio to the oxytetracycline: the catalyst amount is notably high but still halved compared to the starting work reported in the literature [13]. The reactions were performed under MW irradiation at 50 °C for 2 and 4 h. The mixture was then filtered and washed with EtOH/water (1:1) to recover the products from the precipitated complexes.

### 2.3. β-Doxycycline Chacterization

1H-NMR (D_2_O, 600MHz, δ ppm): 7.56, 1H (H-8); 6.96, 1H (H-7); 6.92, 1H (H-9); 3.74, 1H (H-5), 3.40, 1H (H-4); 3.35, 1H (H-6); 3.14, 1H (H-5a); 2.93, 6H (N-CH_3_); 2.77, 1H (H-4a); 1.03, 3H (CH_3_).

13C-NMR from HMQC and HMBC (D_2_O, 150MHz, δ ppm): 135.0 (C-8), 119.5 (C-7), 116.2 (C-9), 67.1 (C-5), 66.3 (C-4), 44.8 (N-CH3), 44.3 (C-5a), 42.0 (C-4a), 33.0 (C-6), 16.1 (CH3).

HPLC-MS, Rt 11.44, *m*/*z* = 445.

### 2.4. α-Doxycycline Chacterization

1H-NMR (D_2_O, 600MHz, δ ppm): 7.32, 1H (H-8); 6.77, 1H (H-7); 6.55, 1H (H-9); 4.2, 1H (H-4); 3.56, 1H (H-5), 2.40, 1H (H-6); 2.30, 1H (H-5a); 2.80, 6H (N-CH_3_); 2.63, 1H (H-4a); 1.30, 3H (CH_3_).

13C-NMR from HMQC and HMBC (D_2_O, 150MHz, δ ppm): 160.0 (C-10), 147 (C-6a) 137.1 (C-8), 116.3 (C-7), 115.9 (C-9), 73.1 (C-12a), 68.4 (C-5), 65.6 (C-4), 44.5 (N-CH_3_), 45.7 (C-5a), 41.3 (C-4a), 37.0 (C-6), 14.9 (CH_3_).

HPLC-MS, Rt 11.56, *m*/*z* = 445.

### 2.5. Molecular Modeling

The X-ray crystal structure of βCD [31] without the crystal water was the starting geometry in the modeling experiments. Oxytetracycline 3D geometry retrieved from PubMed (ID: Conformer3D_CID_54675779, https://pubchem.ncbi.nlm.nih.gov/compound/Oxytetracycline, 31 July 2021), building the presumed intermediates, a reasonable version of RAMEB by the addition of 12 methyl groups, and the creation of molecular ensembles used Jmol (http://www.jmol.org, 31 July 2021).

Molecular mechanics preoptimization of the structures used MMFF94 forcefield implemented in Tinker 8.7.1 (https://dasher.wustl.edu/tinker, 31 July 2021) and heat of formations calculated after PM6 semiempirical optimizations by GAMESS (version gamess-64-2020-R2-pgiblas, https://www.msg.chem.iastate.edu/gamess, 31 July 2021) [32,33,34,35].

## 3. Results and Discussion

### 3.1. A-Doxycycline Synthesis under Heterogeneous Rhodium Catalyst

**Scheme 1:**
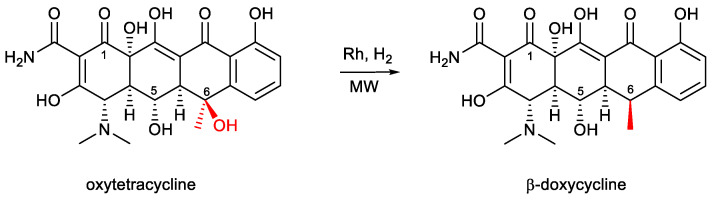
MW-assisted doxycycline synthesis under rhodium heterogeneous catalysis.

Oxytetracycline hydrogenolysis was initially performed without the addition of CDs as chiral selectors over heterogeneous Rh catalysts (Scheme 1). The reactions were performed over commercial Rh/AC and Rh/Al_2_O_3_ 5 wt%: these two commercially available options were chosen to test any role of the support over the final result. DMF/water 1:1 mixture was reported in the literature for the reaction; we also tested methanol (MeOH) and pure water to replace the DMF, aiming for a more sustainable process. The addition of HCl to 0.01 M was used to solubilize the reagent. The list of the experimental conditions tested is presented in Table 1.

Conventional and MW heating were compared because, to the best of our knowledge, MW has never been employed for this synthesis. The results show that it is possible to drastically reduce the reaction time using MW; however, in these conditions only β-doxycycline was obtained. With conventional heating, 16 h were necessary, and β-doxycycline was obtained only in low amounts. Water and methanol are preferred as solvents for the reaction, both in MW-assisted and in conventional experiments, although β-doxycycline was still obtained with DMF/H_2_O under MW. The results were evaluated considering the ratio of doxycycline over oxytetracycline after HPLC-DAD analysis (Graphic 1). β-doxycycline was identified by NMR spectroscopy, also by comparing data with those obtained on a standard of α-doxycycline (see Supplementary Materials: Figures S1–S12).

This improvement in activity under MW is likely due to the interaction of the solid catalyst with the radiation, the faster and homogeneous heating, and possibly the resulting hotspots. The reaction was also tested at 70 °C for 4 h separately in DMF/H_2_O, as a reference from the literature, however, the harsher conditions resulted in a drop in selectivity. Also, a 10:1 substrate to catalyst weight ratio was tested for the optimized conditions over the two supports, but this led to the product only in traces.

Furthermore, the addition of α-doxycycline as an internal standard proved that only the β-form is obtained in these conditions, and so we introduced CDs. As CDs can complex MeOH, it is not recommended to use it as a solvent; otherwise, the complexes with oxytetracycline may be destroyed. Surprisingly, the addition of CDs improved the conversion over Rh/AC when 4 h were used, while yield and selectivity were not affected using Rh/Al_2_O_3_.

The subsequent experiments were all performed in water, using MW and Rh/AC. The first CD test involved the βCD residue in a 2-h reaction. This resulted in α-doxycycline, although in a negligible amount, as confirmed by HPLC-DAD and HPLC-MS analysis with the addition of the internal standard (Figures S13–S15). However, oxytetracycline conversion was not complete. New 4-h hydrogenation was performed, and the α-doxycycline yield improved from 4.0 to 14.1%. This may be explained by the higher stability, and consequent lower reactivity of the complexes, compared to the free oxytetracycline.

Once we had established that CD complexation was able to positively influence the enantioselectivity of the hydrogenation, we ran further tests with different CDs. Moreover, the reaction was performed both on the already-formed complex and with the addition in situ of the native CDs, to investigate which would work the best. The overall results are shown in the following table (Table 2), and selectivity is calculated based on the integrated area ratios:

While both βCD and γCD can complex the substrate, the formation of these adducts is not favored under the reaction conditions, meaning that prior synthesis is recommended. Finally, 3b was the best reaction, showing that the complex is more soluble than its components and has greater stability in water. Doxycycline was easily recovered using the EtOH 50% washes. However, once synthesized, it may be directly recovered and stored in complex form, making for an easier purification step. Complexation with RAMEB, albeit successful, does not lead to the desired compound. α-Doxycycline was identified by NMR spectroscopy comparing data with those obtained on an α-doxycycline standard (Figures S1–S12).

### 3.2. Supposed Mechanism Involved in the Hydrogenation Process

**Scheme 2:**
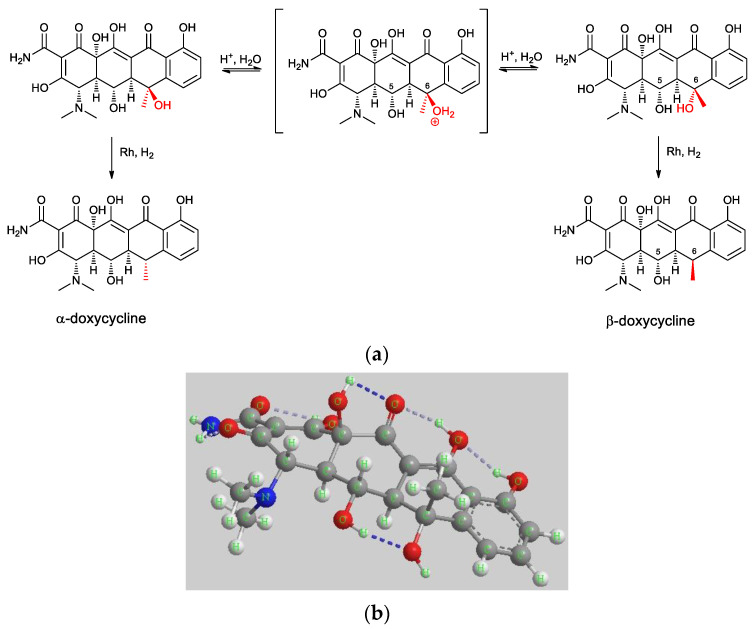
Equilibrium between α and β epimers and following hydrogenation processes (**a**). Hydrogen bond in a six-term cycle that involves the C6 in its β configuration (**b**).

Hydrogenolysis of the C6 benzyl alcohol promoted by the heterogeneous catalyst was previously documented to occur via two possible pathways [36] that can deeply influence the stereoselectivity of the reaction. Having observed different selectivity towards doxycycline epimers when CDs complexes were used, we propose that the one depicted in Scheme 2a is involved in oxytetracycline hydrogenolysis. Oxytetracycline, in an acidic aqueous solution, undergoes epimerization at benzylic carbon C-6. The new structure is highly stabilized by a hydrogen bond formation between the two *cis*-hydroxyl groups in positions 6 and 5, which results in a six-membered ring (Scheme 2b). Further hydrogenation leads to obtaining the β-doxycycline.

In presence of CDs, instead, the interaction between OH in C-6 and the hydroxyl groups of the cyclodextrin cavity competes with that which occurs between the hydroxyls in C-6 and C-5 as a consequence of epimerization.

**Figure 2:**
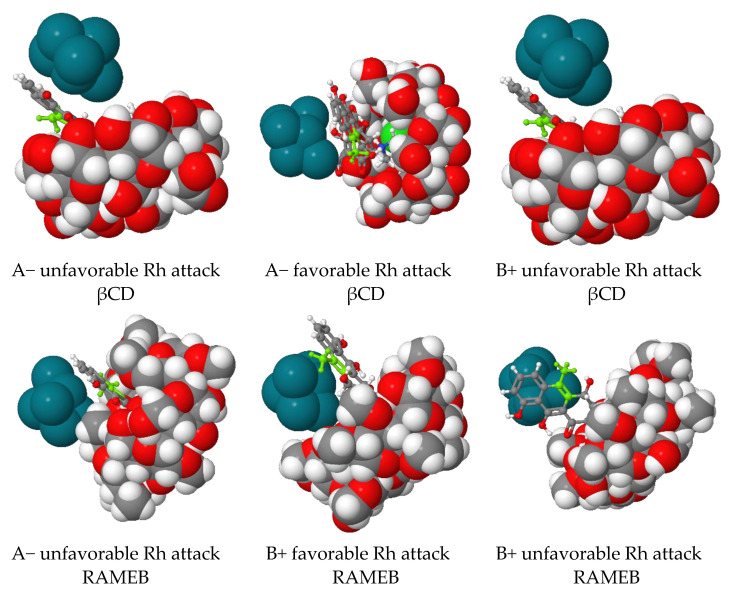
Possible attachment of the Rh nanoparticles around C-6 (figures shows the optimized intermediate/CD complexes (PM6 method) with the molecular mechanics (MMFF94) optimized Rh nanoparticles).

As shown in the energetics in Table 3, the two orientations of tetracycline rings are not equally possible in CD complexes. Both OXY-HCl and the dehydrated intermediate formed in βCD and RAMEB prefer B/C rings in the cavity. The inclusion of A/B rings in this orientation of tetracycline results in significantly lower energy states than C/D inclusion with βCD. The consequence of entrapped C/D rings is that the reducible moiety is not accessible to the hydrogenation catalyst. As the C/D rings move out of the CD cavities, they become virtually freely accessible to the rhodium nanoparticles, but the formation of both C6-epimers of DOXY is not equally possible, as shown in Figure 2. The primary hydroxyl groups are blocking the favorable hydrogenation position. Although both the OXY-HCL and the dehydrated intermediate favor the inclusion complex, in the case of RAMEB, the interaction is as strong as with βCD.

The reverse orientation of the tetracycline ring showed a different picture. Although the encapsulation of the A/B rings is also favored, the complete B/C inclusion of OXY-HCl is weaker in this orientation. While the OXY-HCl prefers to leave the C/D ring from the cavity in the case of RAMEB, the βCD likes to keep close the OXY-HCl to the cavity. The dehydrated intermediate is more stable when the B/C rings are encapsulated; in the case of RAMEB, the energy differences are not significantly different. When the C/D rings are out of the cavity, the hydrophilic ring’s high hydrophilicity and strong H-bond forming ability keep the C/D rings fixed, and the double bond is accessible for the catalyst. Unlike the βCD, the methyl groups of RAMEB can hinder the double bond, more from the unfavorable side, which prevents the formation of DOXYs at all. Although the orientation of the C/D rings of the intermediate toward the primary hydroxyl rim is less favorable, in this case, the methyl substituents of RAMEB can also hinder the unfavorable attack of the Rh nanoparticle to the hydrogenation. The reverse orientation equally allows the hydrogenation from both sides of the tetracycline ring system.

## 4. Conclusions

A simple procedure for the synthesis of α-doxycycline starting from an oxytetracycline/CD complex under hydrogen pressure (5 bar) using heterogeneous catalysis has been described. Under MW irradiation, a 34.0% yield of α-doxycycline was recorded from the oxytetracycline/CD complex. Even though more research is needed to reduce the amount of Rh catalyst needed, we believe that this work should be useful in establishing new means of obtaining α-doxycycline selectively, as well as contributing to the future design of even more sustainable organic synthetic procedures based on heterogeneous catalysis in combination with MW irradiation and green solvents.

## Figures and Tables

**Table 1:** Doxycycline synthesis under conventional and MW-assisted conditions.

Entry	Heating	Solvent	Catalyst	H_2_ (bar)	T (°C)	Time (h)	Yield β-Doxy (%)	Yield α-Doxy (%)
1a	Conventional ^a^	DMF/H_2_O	Rh/AC 5%	5	50	16	*traces*	n.d.
		DMF/H_2_O	Rh/AC 5%	5	70	16	*traces*	n.d.
2a		H_2_O	Rh/AC 5%	10	50	16	*traces*	n.d.
3a		MeOH	Rh/AC 5%	10	50	16	3.00	n.d.
4a		H_2_O	Rh/Al_2_O_3_ 5%	10	50	16	0.50	n.d.
5a		MeOH	Rh/Al_2_O_3_ 5%	10	50	16	*traces*	n.d.
6a	MW ^b^	DMF/H_2_O	Rh/AC 5%	5	50	4	1.20	n.d.
7a		DMF/H_2_O	Rh/AC 5%	5	50	2	2.15	n.d.
8a		DMF/H_2_O	Rh/AC 5%	5	70	2	0.95	n.d.
9a		H_2_O	Rh/AC 5%	5	50	2	*traces*	n.d.
10a		MeOH	Rh/AC 5%	5	50	2	36.90	n.d.
11a		H_2_O	Rh/Al_2_O_3_ 5%	5	50	2	34.20	n.d.
12a		MeOH	Rh/Al_2_O_3_ 5%	5	50	2	1.10	n.d.

^a^ conventional heating, 50 mg oxytetracycline, 5 mg Rh/AC 5 wt%. ^b^ MW heating, 50 mg oxytetracycline, 50 mg Rh/AC 5 wt%. n.d. = non detected.

**Table 2:** MW-assisted synthesis of doxycycline starting from oxytetracycline/β-CD complex.

Entry	Substrate	Conversion (%)	Yield α-Doxy (%)	Yield β-Doxy (%)
1b	βCD solid ^a^	44.3	4.0	16.1
2b	βCD solid ^b^	60.0	14.1	32.2
3b	βCD filtrate ^b^	100	34.0	n.d.
4b	βCD in situ ^b^	100	19.1	36.2
5b	RAMEB ^b^	68.3	n.d.	n.d.
6b	γCD ^b^	100	26.2	14.3
7b	γCD in situ ^b^	100	5.2	6.7

^a^ 50 mg oxytetracycline, 50 mg Rh/AC 5 wt%, 2 mL H_2_O, 50 °C, 5 bar H_2_, 2 h. ^b^ 50 mg oxytetracycline, 50 mg Rh/AC 5 wt%, 2 mL H_2_O, 50 °C, 5 bar H_2_, 4 h. n.d. = non detected.

**Table 3:** The heat of formation differences (Kcal/mol) of the complete and incomplete inclusion compounds between CDs and oxytetracycline, relative to the free components.

	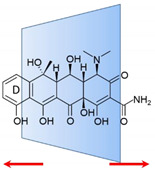			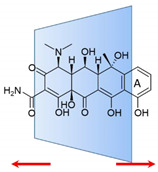		
	A−	Ai	A+	B−	Bi	B+
βCD/OXY-HCl	−38.6	−40.6	−31.2	−14.0	−25.2	−25.2
RAMEB/OXY-HCl	−34.7	−58.4	−25.6	−17.3	−19.0	−39.6
βCD/Dehydrated-HCl	−23.6	−20.2	−20.0	−13.2	−32.4	−22.8
RAMEB/Dehydrated-HCl	−39.3	−50.7	−24.8	−32.2	−32.0	−29.2

## Data Availability

Not applicable.

## Supplementary Materials

The following are available online at https://www.mdpi.com/article/10.3390/antibiotics10091084/s1. Figure S1: 1H-NMR spectrum of reaction products obtained in presence of β-cyclodextrin. Most abundant signals are attributed to α-doxycycline, signals labelled with * are those of methanol, whereas signals identified with # are relative to different isomers (β-doxycycline and, probably, 4-epidoxycycline). 600 MHz, D_2_O, RT. Figure S2: 1H-NMR spectrum of α-doxycycline reference compound (technical grade). signals labelled with * are those of methanol, whereas signals identified with # are relative to different isomer (β-doxycycline). 600 MHz, D_2_O, RT. Figure S3: DQF-COSY spectrum of reaction products obtained in presence of β-cyclodextrin. Figure S4: 1H-13C-HMQC spectrum of reaction products obtained in presence of β-cyclodextrin. Figure S5: 1H-13C-HMBC spectrum of reaction products obtained in presence of β-cyclodextrin. Figure S6: DQF-COSY spectrum of α-doxycycline reference compound. Figure S7: 1H-13C-HMQC spectrum of α-doxycycline reference compound. Figure S8: 1H-13C-HMBC spectrum of α-doxycycline reference compound. Figure S9: 1H-NMR spectrum of β-doxycycline (reaction products obtained without β-cyclodextrin). Figure S10: COSY spectrum of β-doxycycline (reaction products obtained without β-cyclodextrin). Figure S11: 1H-13C HMQC spectrum of β-doxycycline (reaction products obtained without β-cyclodextrin). Figure S12: 1H-13C HMBC spectrum of β-doxycycline (reaction products obtained without β-cyclodextrin). Figure S13: Example of HPLC-MS trace of a reaction product obtained without CD addition (trace “a” total ion current, trace “b” extraction of ion with m/z = 445), before and after (trace “c” total ion current, trace “d” extraction of ion with m/z = 445) the addition of α-doxycycline reference compound. Figure S14: HPLC-MS trace of α-doxycycline reference material (trace “a” total ion current, trace “b” extraction of ion with m/z = 445). Figure S15: Example of HPLC-MS trace of reaction product obtained in presence of β-cyclodextrin (trace “a” total ion current, trace “b” extraction of ion with m/z = 445), before and after (trace “c” total ion current, trace “d” extraction of ion with m/z = 445) the addition of α-doxycycline reference compound.
