# Supplementary material for: Microwave-Assisted, One-Pot Synthesis of Doxycycline under Heterogeneous Catalysis in Water
Source: Antibiotics (Basel). 2021 Sep 8;10(9):1084. doi: 10.3390/antibiotics10091084 (PMC8466421; doi:10.3390/antibiotics10091084)
Supplement: Supplementary file 1 [file antibiotics-10-01084-s001.zip › antibiotics-1354095-supplementary.pdf]

Supplementary Materials

Microwave-assisted, one-pot synthesis of Doxycycline under heterogeneous catalysis in water

Fabio Bucciol[1], Elia Maffeis[2], Emanuela Calcio Gaudino[1], László Jicsinszky[1], Silvia Tagliapietra[1], Alessandro Barge[1], Cristina Prandi[3] and Giancarlo Cravotto[1]*

[1] Department of Drug Science and Technology, University of Turin, Via P. Giuria 9, 10125 Turin, Italy; fabio.bucciol@unito.it (F.B.); emanuela.calcio@unito.it (E.C.G.); laszlo.jicsinszky@unito.it (L. J.); silvia.tagliapietra@unito.it (S.T.); alessandro.barge@unito.it (A.B.)
[2] Huvepharma Italia Srl, Via Roberto Lepetit, 142, 12075 Garessio (CN) Italy; elia.maffeis@unito.it
[3] Department of Chemistry, University of Turin, Via P. Giuria 7, 10125 Turin, Italy; cristina.prandi@unito.it
* Correspondence: giancarlo.cravotto@unito.it; Tel.: +39-011-6707183

NMR characterization

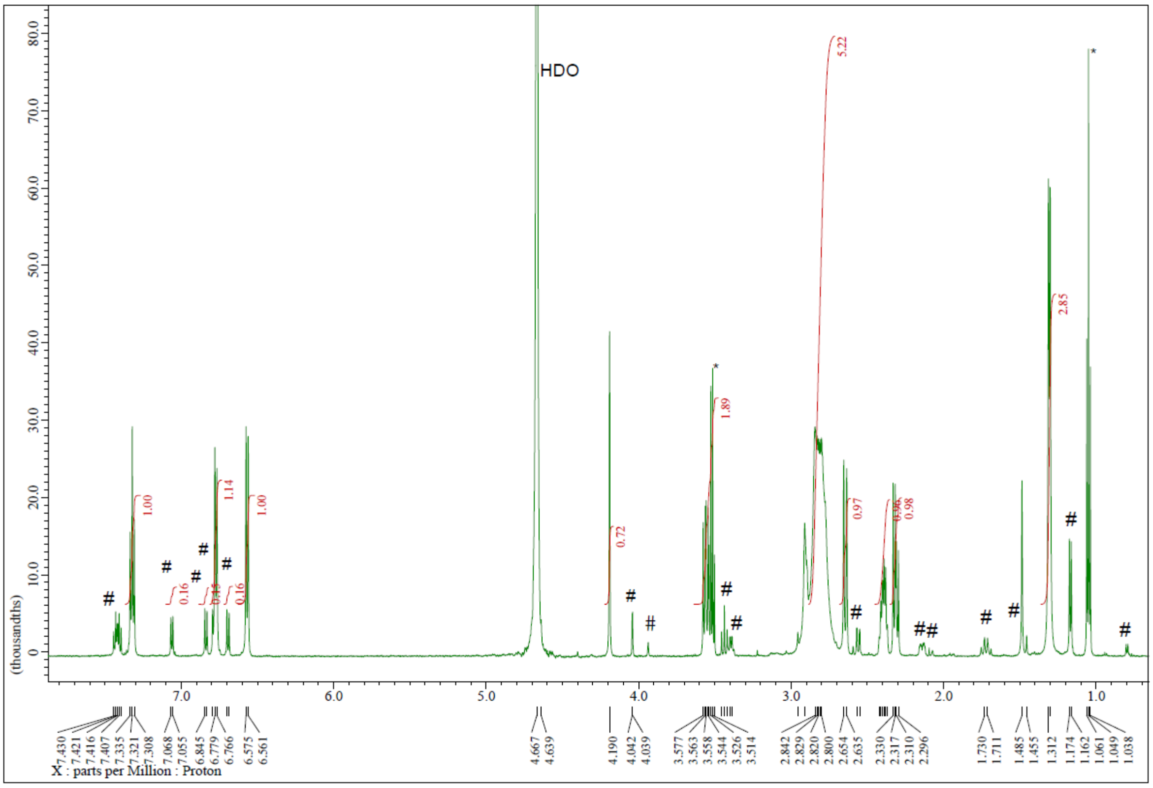

Figure S1. 1H-NMR spectrum of reaction products obtained in presence of β-cyclodextrin. Most abundant signals are attributed to α-doxycycline, signals labelled with * are those of methanol, whereas signals identified with # are relative to different isomers (β-doxycycline and, probably, 4-epidoxycycline). 600MHz, D_2O, RT.

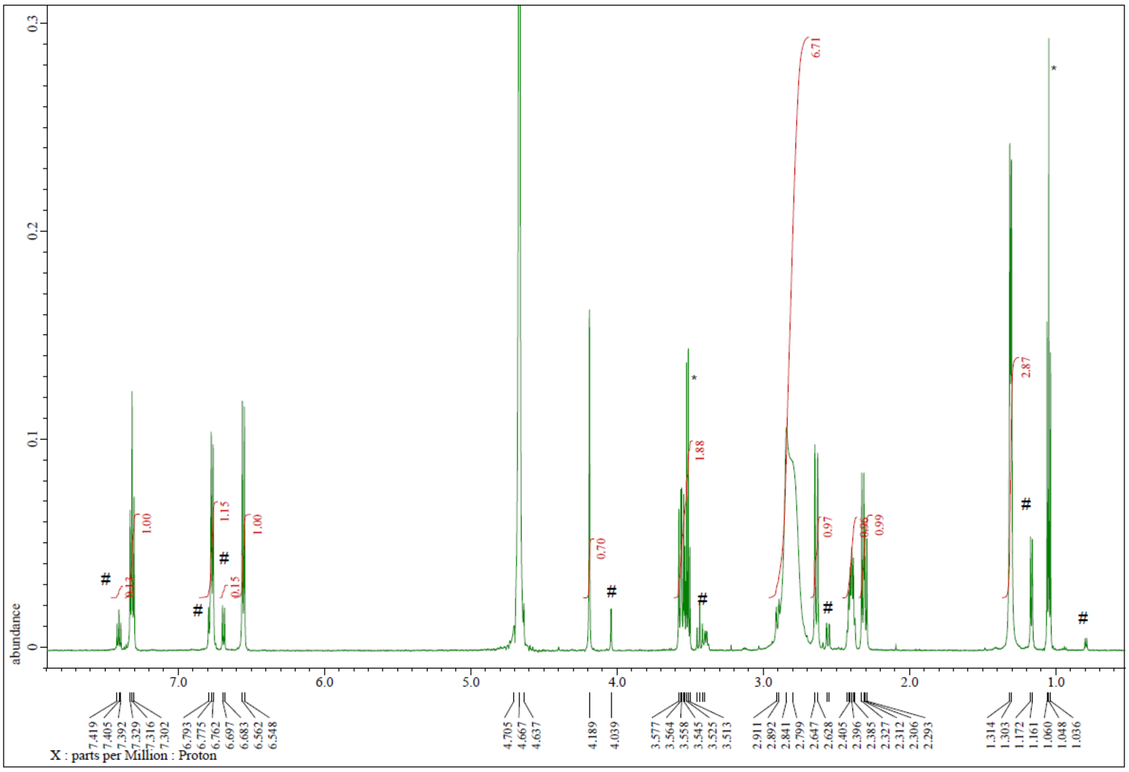

Figure S2. 1H-NMR spectrum of α-doxycycline reference compound (technical grade). signals labelled with * are those of methanol, whereas signals identified with # are relative to different isomer (β-doxycycline). 600MHz, D_2O, RT.

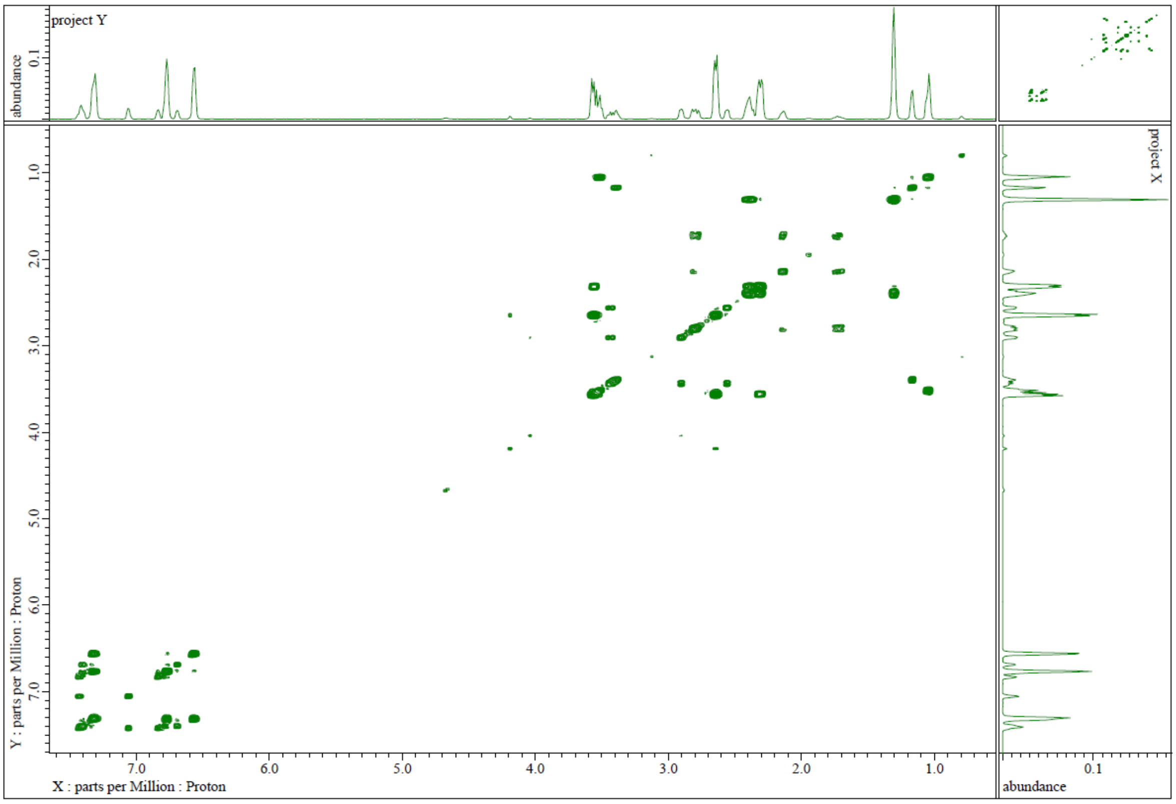

Figure S3. DQF-COSY spectrum of reaction products obtained in presence of β-cyclodextrin.

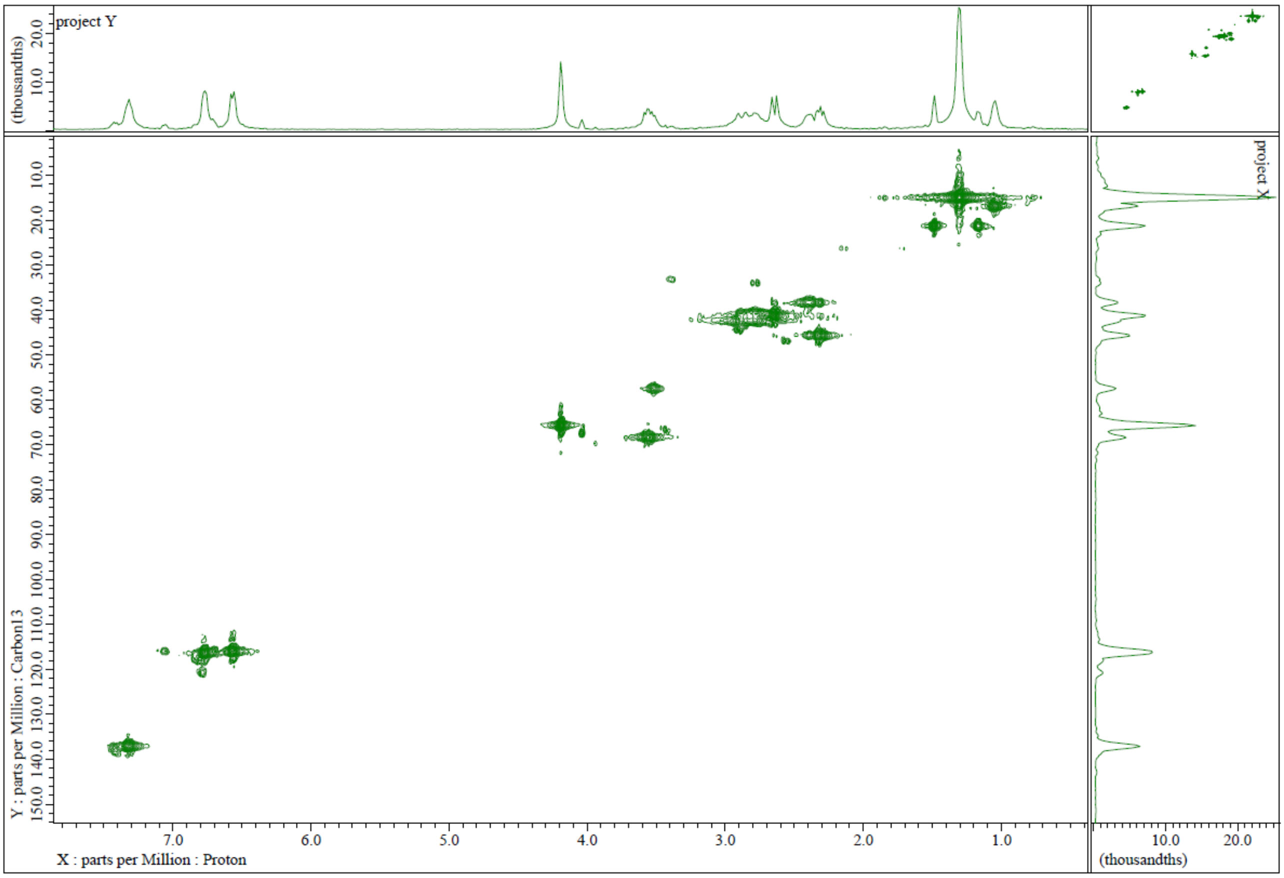

Figure S4. 1H-13C-HMQC spectrum of reaction products obtained in presence of β-cyclodextrin.

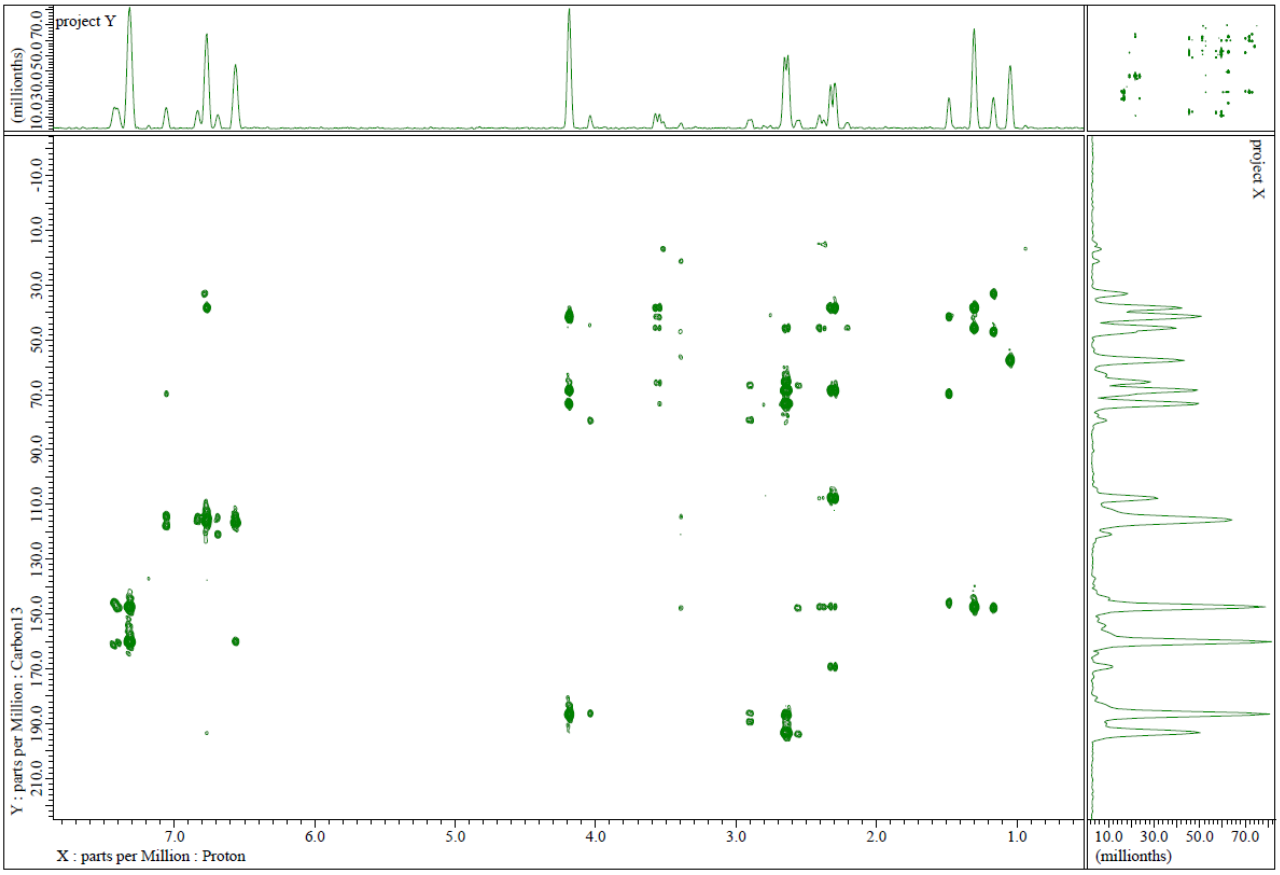

Figure S5. 1H-13C-HMBC spectrum of reaction products obtained in presence of β-cyclodextrin.

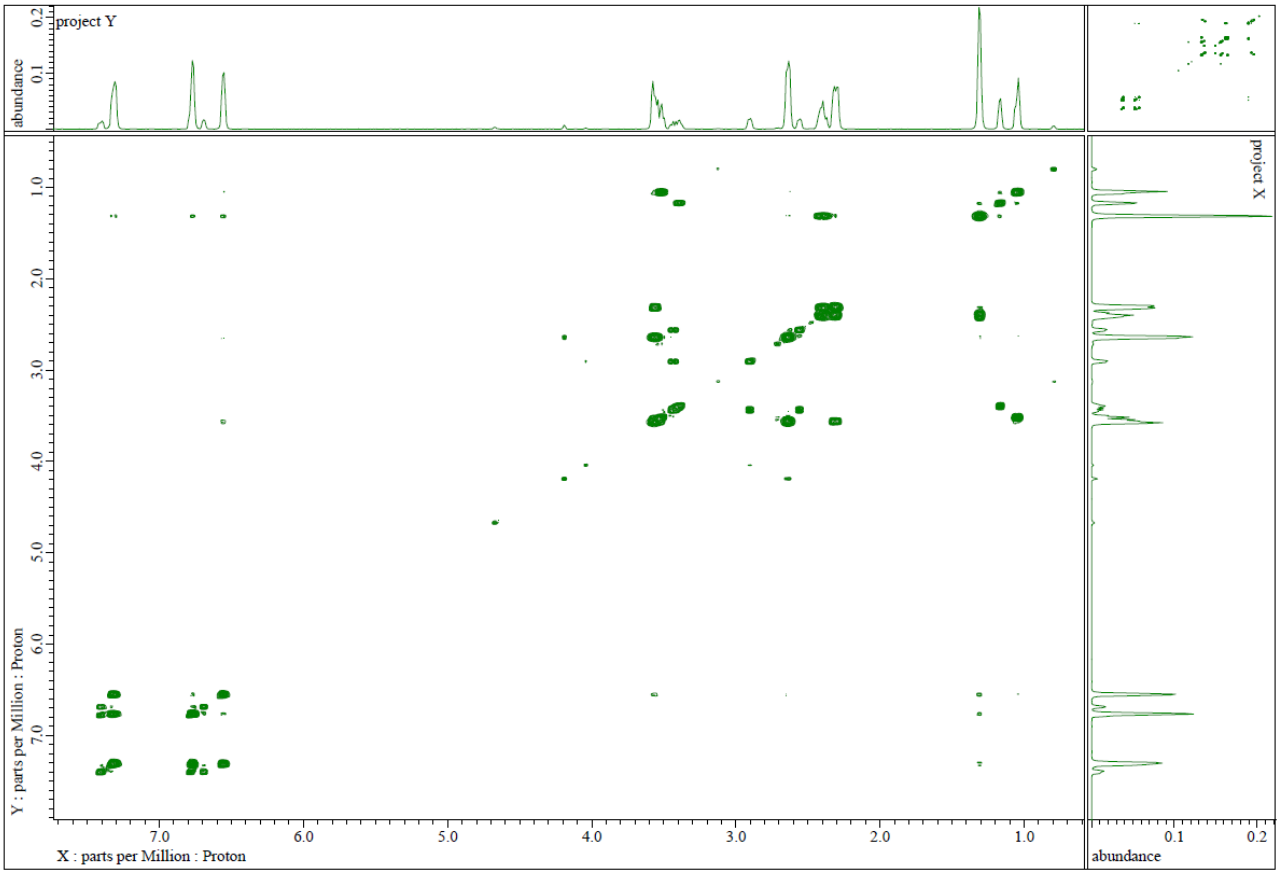

Figure S6. DQF-COSY spectrum of α-doxycycline reference compound.

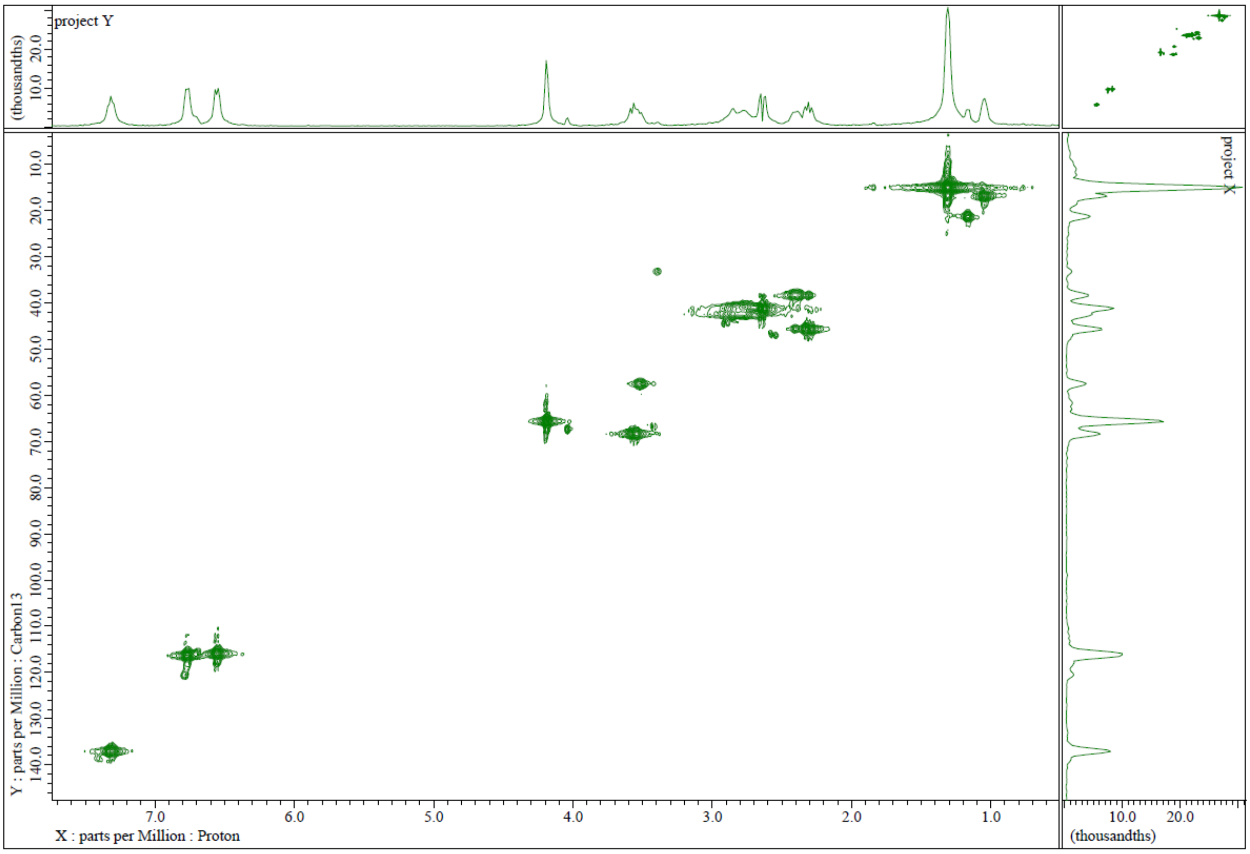

Figure S7. 1H-13C-HMQC spectrum of α-doxycycline reference compound.

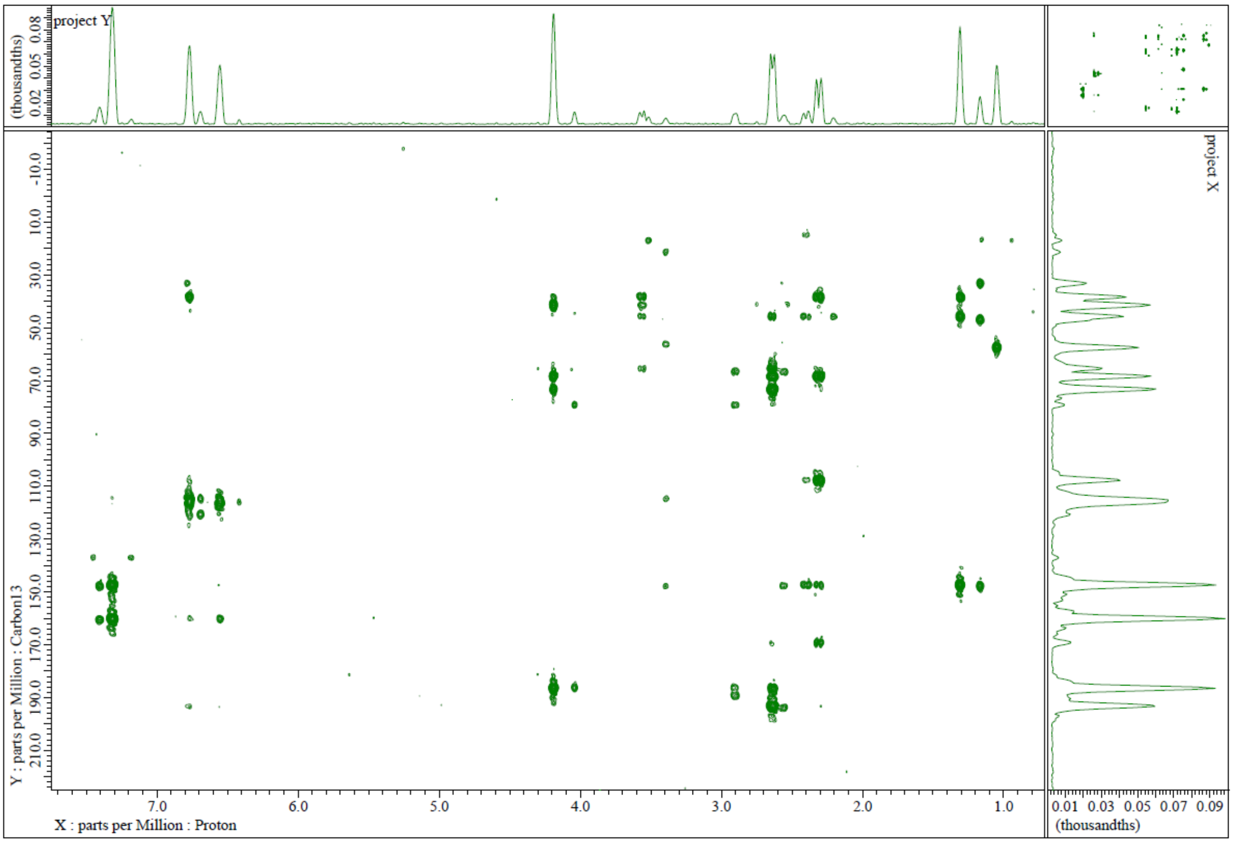

Figure S8. 1H-13C-HMBC spectrum of α-doxycycline reference compound.

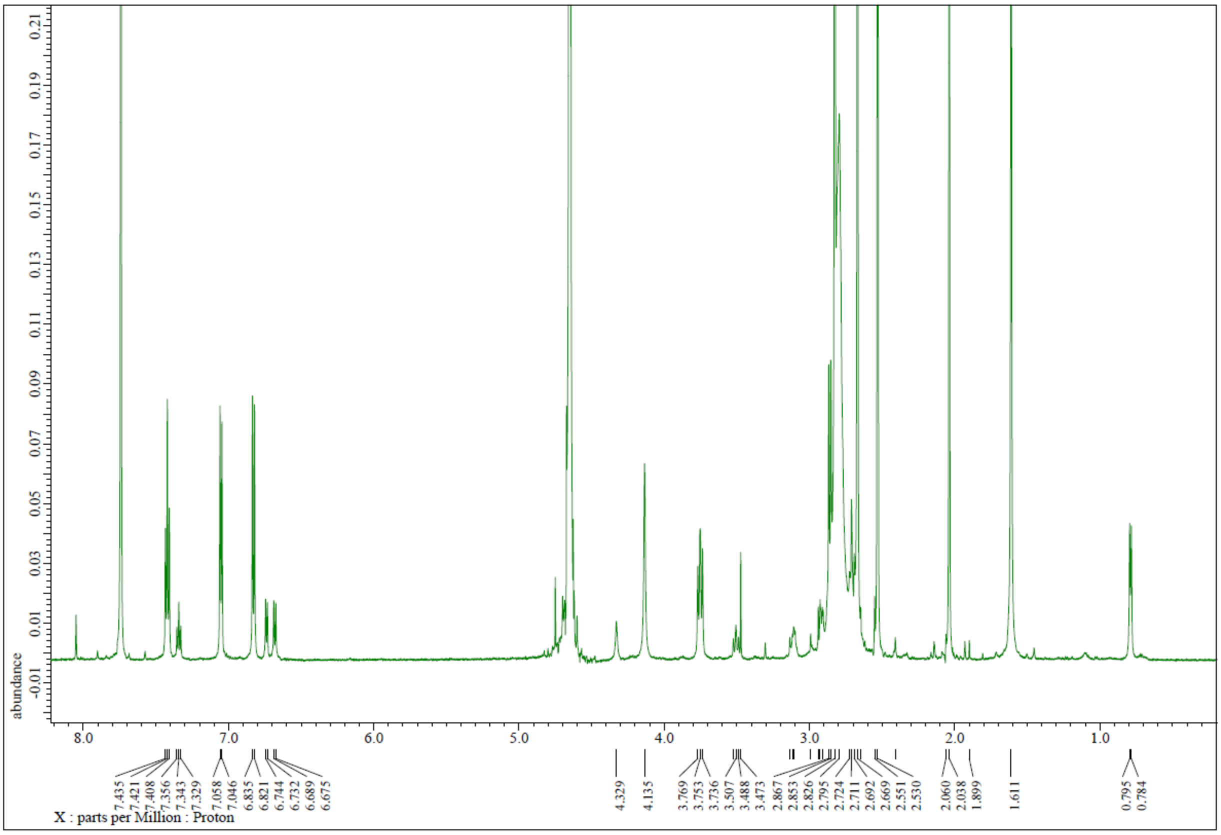

Figure S9. 1H-NMR spectrum of β-doxycycline (reaction products obtained without β-cyclodextrin).

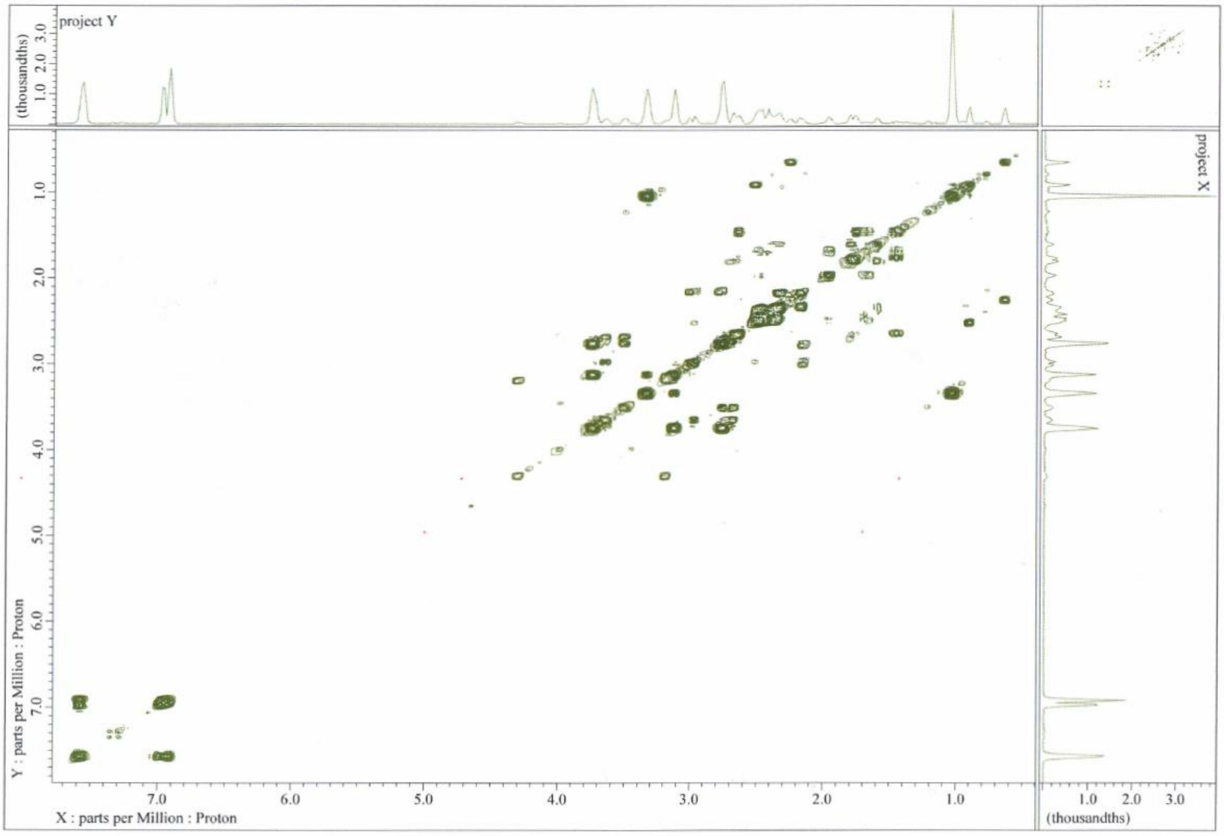

Figure S10. COSY spectrum of β-doxycycline (reaction products obtained without β-cyclodextrin).

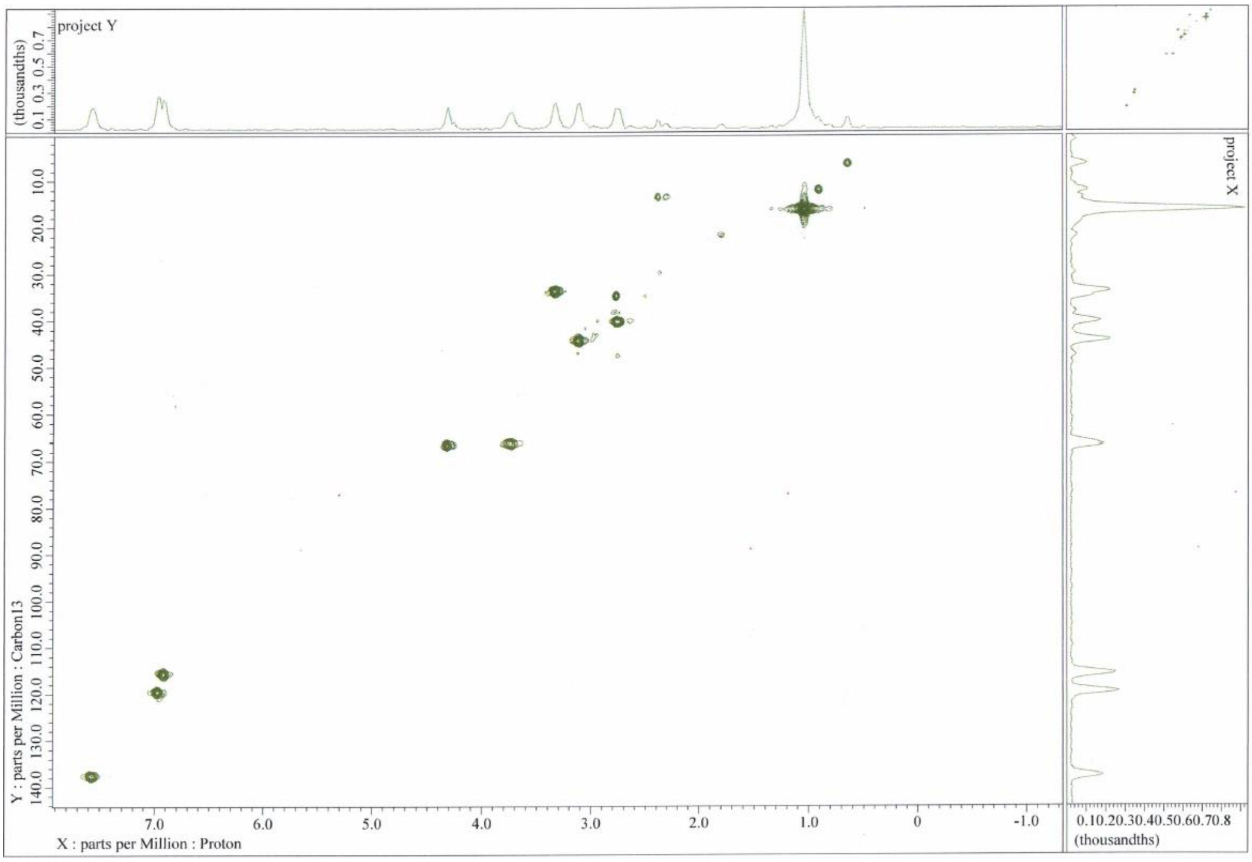

Figure S11. 1H-13C HMQC spectrum of β-doxycycline (reaction products obtained without β-cyclodextrin).

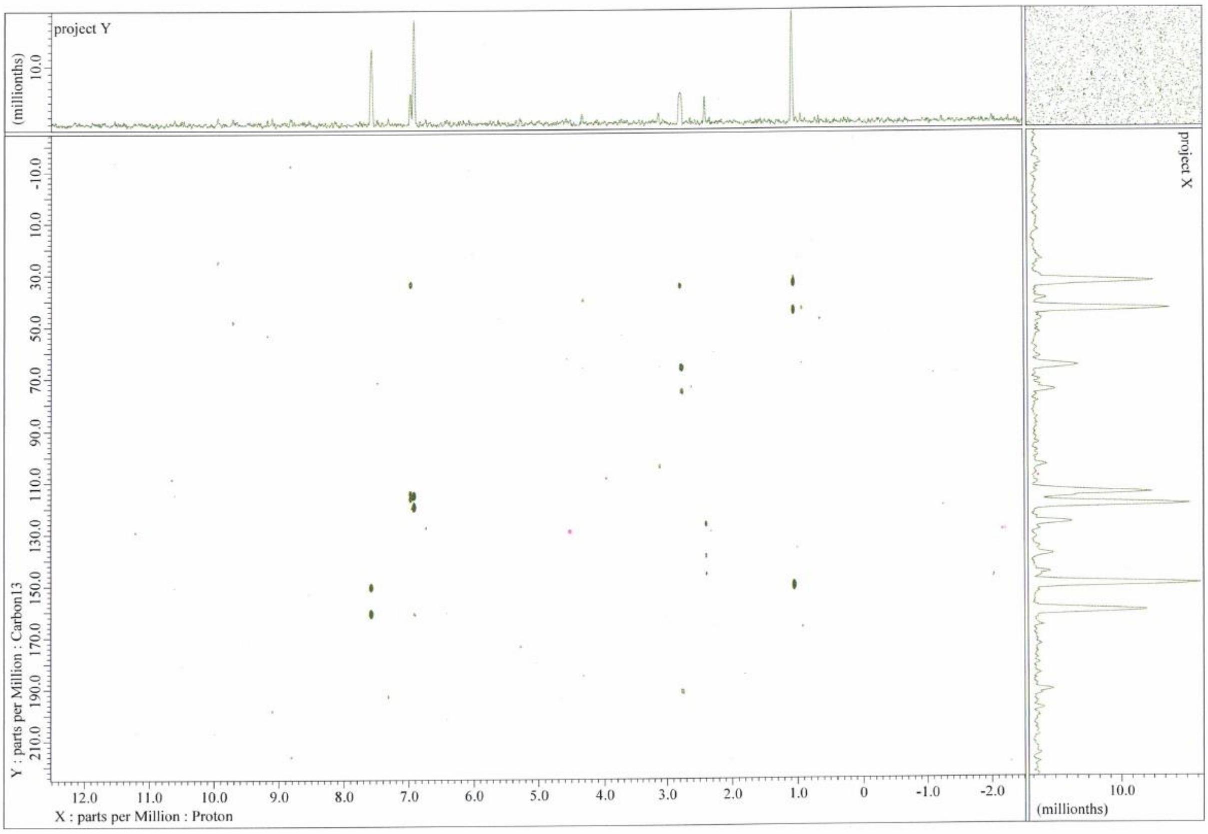

Figure S12. 1H-13C HMBC spectrum of β-doxycycline (reaction products obtained without β-cyclodextrin).

HPLC-MS analysis

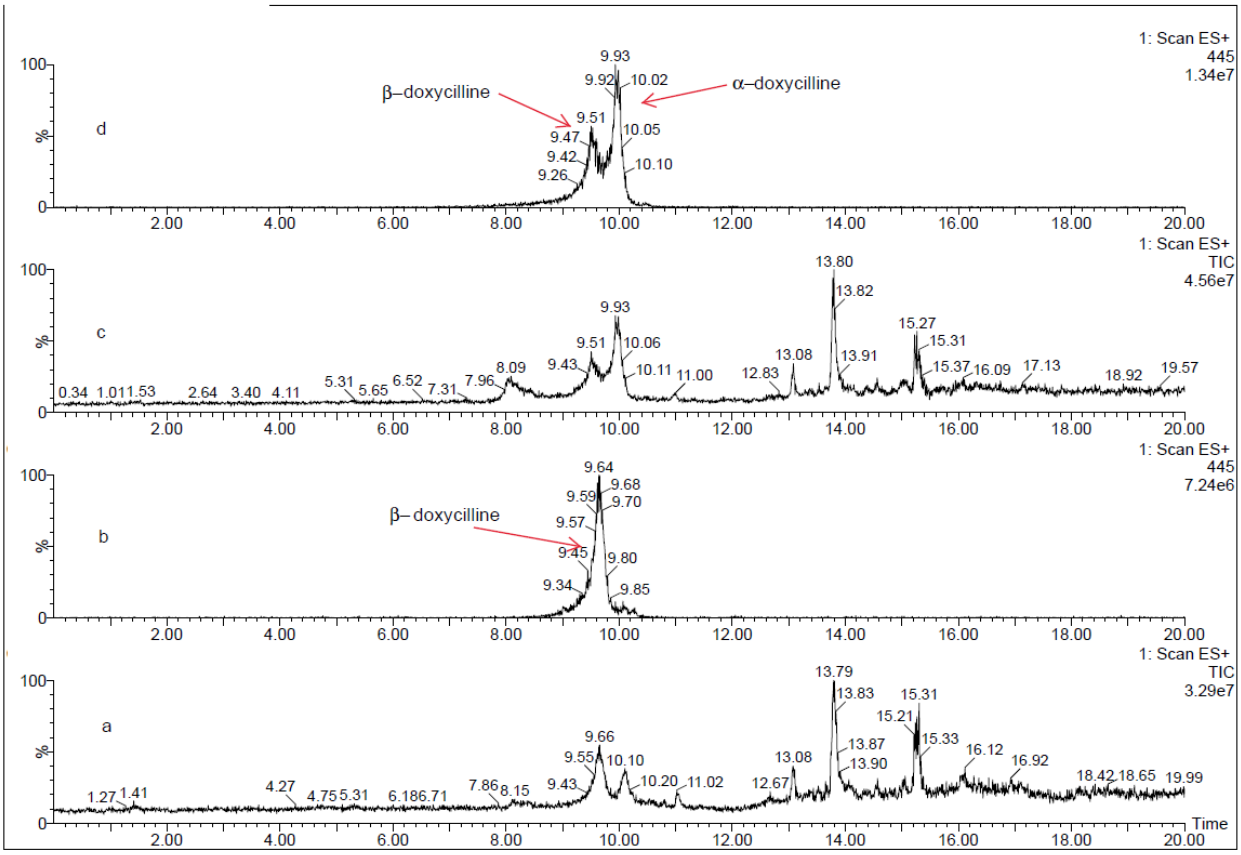

Figure S13. Example of HPLC-MS trace of a reaction product obtained without CD addition (trace "a" total ion current, trace "b" extraction of ion with m/z= 445), before and after (trace "c" total ion current, trace "d" extraction of ion with m/z= 445) the addition of α-doxycycline reference compound.

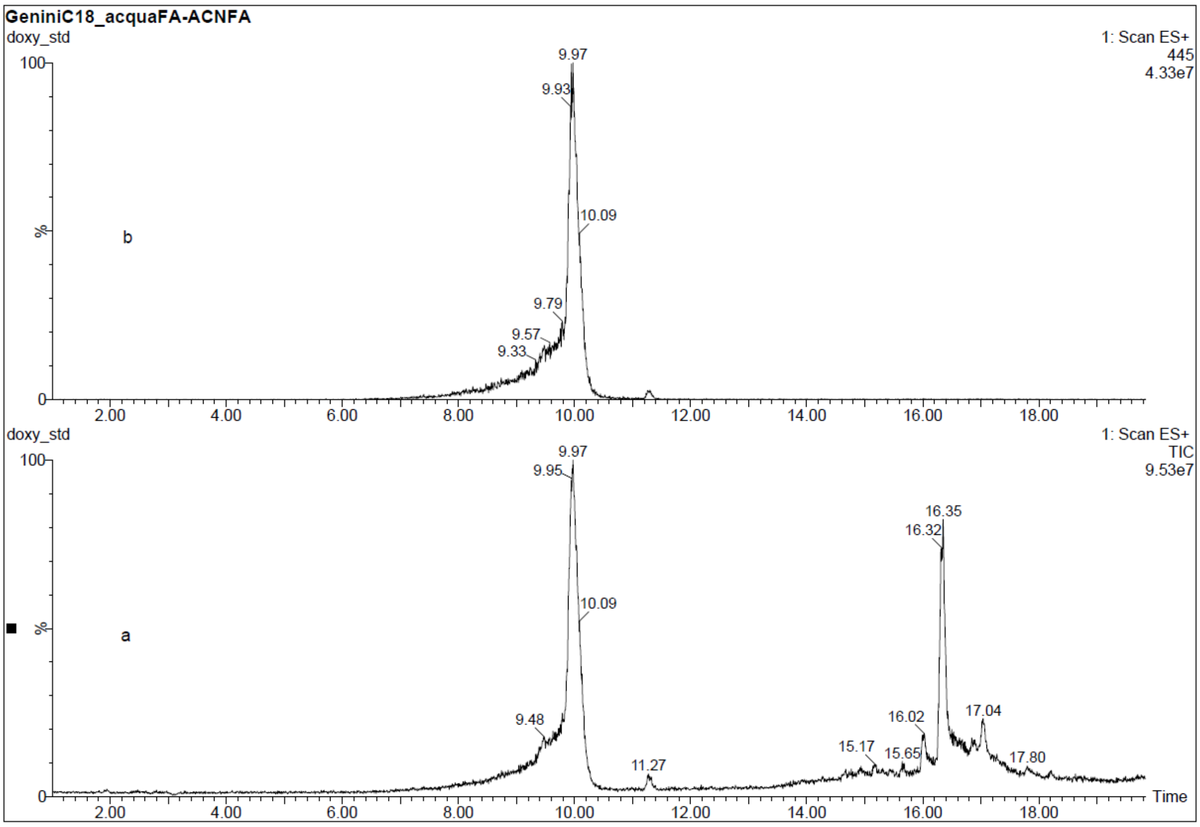

Figure S14. HPLC-MS trace of α-doxycycline reference material (trace "a" total ion current, trace "b" extraction of ion with m/z= 445).

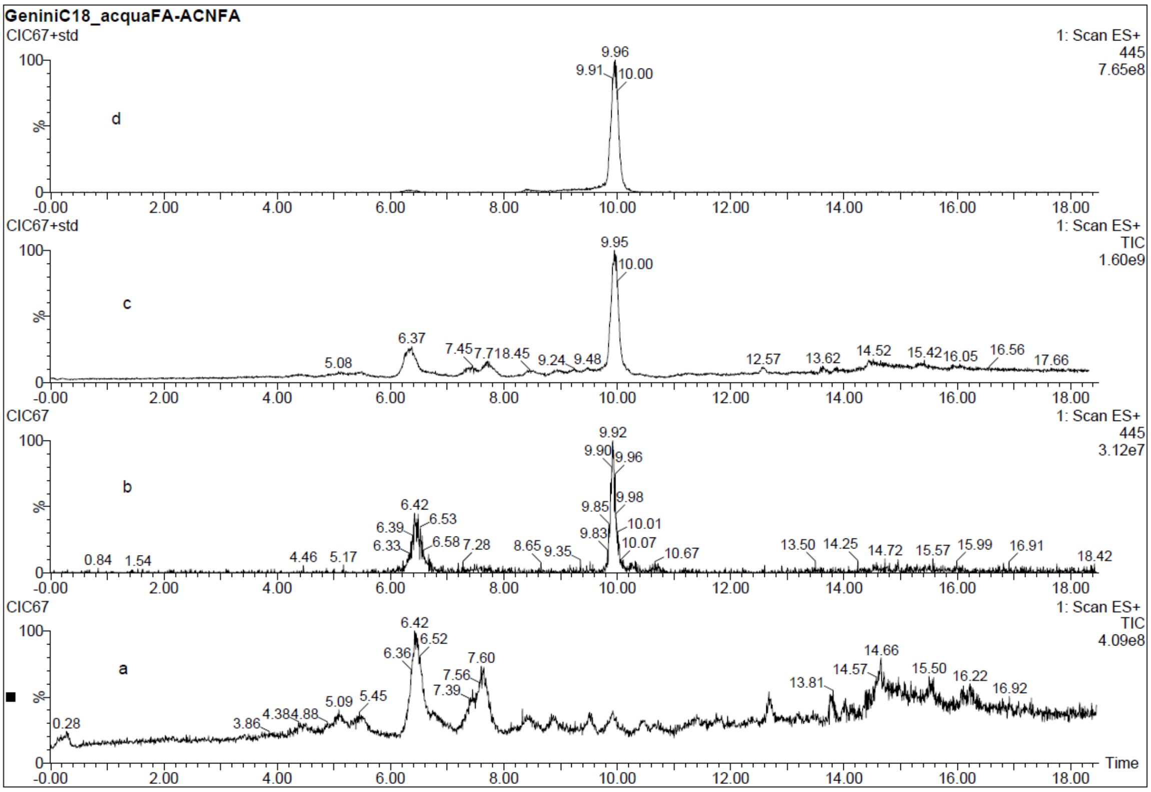

Figure S15. Example of HPLC-MS trace of reaction product obtained in presence o cyclodextrin (trace "a" total ion current, trace "b" extraction of ion with m/z= 445), before and after (trace "c" total ion current, trace "d" extraction of ion with m/z= 445) the addition of α-doxycycline reference compound.
